# Draft Genome Sequence of Aldehyde-Degrading Strain *Halomonas axialensis* ACH-L-8

**DOI:** 10.1128/genomeA.00287-16

**Published:** 2016-04-14

**Authors:** Jun Ye, Chong Ren, Xiexie Shan, Runying Zeng

**Affiliations:** a College of Food and Biological Engineering, Jimei University, Xiamen, China; b State Key Laboratory Breeding Base of Marine Biogenetic Resource, Third Institute of Oceanography SOA, Xiamen, China; c South China Sea Bio-Resource Exploitation and Utilization Collaborative Innovation Center, Xiamen, China; d China Shipbuilding Industry Corporation (CSIC) No. 710 Institute, Wuhan, China

## Abstract

*Halomonas axialensis* ACH-L-8, a deep-sea strain isolated from the South China Sea, has the ability to degrade aldehydes. Here, we present an annotated draft genome sequence of this species, which could provide fundamental molecular information on the aldehydes-degrading mechanism.

## GENOME ANNOUNCEMENT

*Halomonas axialensis* was first isolated by Kaye et al. (1) from low-temperature hydrothermal fluid (Cloud Vent) at 1530-m depth. They are rod-shaped with rounded ends and grow in psychrotolerant and moderately halophilic environments. Gutierrez et al. (2) found *Halomonas* sp. strain MCTG39a can degrade hydrocarbon. On 12 March 2014, we isolated *H. axialensis* ACH-L-8 from deep sea water (2000-m depth) from the South China Sea that can grow with acetaldehyde as the sole carbon source and degrade aldehydes. This strain has been deposited in the Marine Culture Collection of China (accession number MCCC 1A10756). The 16S rRNA sequence of ACH-L-8 available in the GenBank database (accession number KP689598) showed 99.44% identity with *H. axialensis* Althf1^T^ (accession number NR_027219.1) (1).

Whole-genome sequencing was performed using Illumina HiSeq 2000 technology by generating Illumina paired-end (PE) libraries (300 bp length) at the Shanghai Majorbio Bio-pharm Technology Co., Ltd. (Shanghai, China). Approximately 4,423,238 × 2 Clean data pair reads with 94.84% Q20, which provided a 239-fold depth of coverage, were first assembled using SOAPdenovo version 2.04 (http://soap.genomics.org.cn). The coding sequences (CDS) were predicted using Glimmer version 3.0.2 (http://ccb.jhu.edu/software/glimmer/index.shtml). Homologous comparison of all the genes was performed by BLAST against the NCBI nonredundant public database and KEGG database (3). The rRNAs and tRNAs were identified using Barrnap version 0.4.2 (http://www.vicbioinformatics.com/software.barrnap.shtml) and tRNAscan-SE v1.3.1 (http://lowelab.ucsc.edu/tRNAscan-SE/).

The genome of *H. axialensis* ACH-L-8 consists of 122 contigs (>1000 bp), with a total length of 3592820 bp and a G+C content of 56.85%; the largest contig length is 164679 bp; the N_50_ contig length is 70749 bp. There are 3352 predicted open reading frames (ORFs), 51 tRNA genes, and 3 rRNA clusters. The results based on KEGG database analysis predicted that *H. axialensis* ACH-L-8 contains 20 ALDH (aldehyde dehydrogenase) encoding genes that are involved in 14 metabolic pathways associated with aldehydes degradation. ALDH activity plays an important role in the process of degrading aldehydes. ALDHs are NAD(P)^+^-dependent enzymes, which catalyze the oxidation of a broad variety of aldehydes to their corresponding acids (4). Although aldehydes could be formed endogenously during the metabolism of amino acids, vitamins, lipids, and carbohydrates (5), they are cytotoxic and mutagenic compounds in the environments as a by-product of industrial activities (6). The *H. axialensis* genome sequence may provide fundamental molecular information on the aldehydes-degrading mechanism. It is important to further study ALDH genes in strain *H. axialensis* ACH-L-8, in order to promote its application in the treatment of aldehyde-polluted environments.

### Nucleotide sequence accession numbers.

This whole-genome shotgun project has been deposited at DDBJ/EMBL/GenBank under the accession number JYFV00000000. The version described in this paper is version JYFV01000000.
